# PrimPol-deficient cells exhibit a pronounced G2 checkpoint response following UV damage

**DOI:** 10.1080/15384101.2015.1128597

**Published:** 2015-12-22

**Authors:** Laura J. Bailey, Julie Bianchi, Nadia Hégarat, Helfrid Hochegger, Aidan J. Doherty

**Affiliations:** a Genome Damage and Stability Centre, School of Life Sciences, University of Sussex, Brighton, UK; b Present address: Department of Oncology-Pathology, Cancer Center Karolinska, Karolinska Institutet, Stockholm, Sweden

**Keywords:** cell cycle, checkpoint, Chk1, DT40, PrimPol, polymerase, primase, replication, UV, TLS

## Abstract

PrimPol is a recently identified member of the archaeo-eukaryote primase (AEP) family of primase-polymerases. It has been shown that this mitochondrial and nuclear localized enzyme plays roles in the maintenance of both unperturbed replication fork progression and in the bypass of lesions after DNA damage. Here, we utilized an avian (DT40) knockout cell line to further study the consequences of loss of PrimPol (*PrimPol*^*−/−*^) on the downstream maintenance of cells after UV damage. We report that *PrimPol*^*−/−*^ cells are more sensitive to UV-C irradiation in colony survival assays than Pol η-deficient cells. Although this increased UV sensitivity is not evident in cell viability assays, we show that this discrepancy is due to an enhanced checkpoint arrest after UV-C damage in the absence of PrimPol. *PrimPol*^*−/−*^ arrested cells become stalled in G2, where they are protected from UV-induced cell death. Despite lacking an enzyme required for the bypass and maintenance of replication fork progression in the presence of UV damage, we show that *PrimPol*^*−/−*^ cells actually have an advantage in the presence of a Chk1 inhibitor due to their slow progression through S-phase.

## Introduction

Genomic DNA is constantly under attack from a range of damaging agents that induce lesion formation, which can obstruct the replication machinery. Ultra-violet (UV) light generates DNA photoproducts, such as cyclopyrimidine dimers (CPDs) and 6-4 photoproducts (6-4pps), that can prove lethal to cells as they disrupt DNA replication and transcription processes.^1^ In addition, endogenous DNA structures, such as G4 quadruplexes, can also form replicase-stalling obstacles.^2^ These barriers must be overcome in order to produce a faithful copy of the entire genome to pass on to the daughter cell. Eukaryotic cells possess a number of mechanisms to restart stalled forks. These include dormant origin firing, homologous recombination (HR) and specialized DNA polymerases involved in trans-lesion synthesis (TLS) bypass of replication-stalling lesions.^3-5^ These polymerases include Pol η, which can bypass CPDs, as well as Pol ζ, Pol κ, Pol ι and Rev1.

Recently, a novel primase-polymerase called PrimPol has been identified in eukaryotic cells, which also has the ability to bypass lesions, including UV photoproducts. PrimPol is a a member of the archaeo-eukaryotic primase (AEP) superfamily^6^ and, like some other AEPs, is capable of DNA-dependent RNA/DNA priming and DNA-dependent DNA synthesis.^7^ PrimPol is localized in both the nucleus and mitochondrion, where it plays roles in damage tolerance during DNA replication.^8-14^ PrimPol can replicate directly across 6-4pps and oxidative lesions and thus its polymerase activities may allow it to synthesize directly opposite lesions to maintain fork progression. We previously demonstrated an increase in replication stalling in *PrimPol*^*−/−*^ cells after the induction of UV-C lesions and an increased sensitivity to UV-C damage when PrimPol is depleted in a *Pol* η^*−/−*^ background.^8^ *PrimPol*^*−/−*^ cells also exhibit reduced fork rates in the absence of damage and depletion of a PrimPol ortholog in trypanosomes is lethal.^15^ These reports suggest that PrimPol may also be required to assist in the replication of undamaged templates that are “difficult” to replicate, a role currently ascribed to other TLS polymerases or the HR machinery.^16-18^ PrimPol's dual activities as a DNA primase and polymerase suggest that it may also play a number of additional roles. Repriming has been demonstrated to restart replication in *E. coli*^19,20^ and has also been proposed to occur in eukaryotic cells.^21-23^ Notably in this regard, PrimPol's primase activity has been implicated in DNA damage tolerance following UV-C damage. It has been reported that PrimPol's primase activity appears to be required for re-priming downstream of replication blocking DNA lesions, thus facilitating progression of replication on UV-C damaged templates.^10,12^ PrimPol is a mutagenic polymerase that displays an insertion-deletion (indel) error signature.^24^ Human PrimPol interacts with the single-strand binding proteins (SSBs), mtSSB and RPA, binding directly to the N-terminal domain of RPA70.^24^ SSBs appear to regulate PrimPol's polymerase and primase activities and it may, in addition to recruiting the enzyme, regulate synthesis by this enzyme at stalled forks and modulate its mutagenic potential during replication restart.

Here, we report the damage sensitivity and cell cycle progression defects associated with avian DT40 cells deleted for PrimPol (*PrimPol*^*−/−*^) grown in the presence or absence of UV-C damage. We have identified that these cells are significantly more sensitivity to UV damage than previously reported, revealing that *PrimPol*^*−/−*^ cells are even more sensitive to UV-C damage than even *Pol*η^*−/−*^ cells in colony formation assays. An extended G2 arrest and decreased apoptosis is also evident in *PrimPol*^*−/−*^ cells after exposure to high fluences of UV-C irradiation. In addition, we also identified a resistance to G2 checkpoint inhibitors in these cells. Together, these findings suggest that in the absence of PrimPol, cells are unable to sufficiently bypass / repair damage caused by UV-C. This results in an extended G2 arrest that, in many cases, appears to be inescapable. However, the decreased rates of replication and cell cycle progression observed in the absence of PrimPol appears to have an unexpected protective effect that limits UV-induced cell death.

## Results

### *PrimPol*^*−/−*^ cells fail to proliferate after UV-C damage

**Figure 1.**
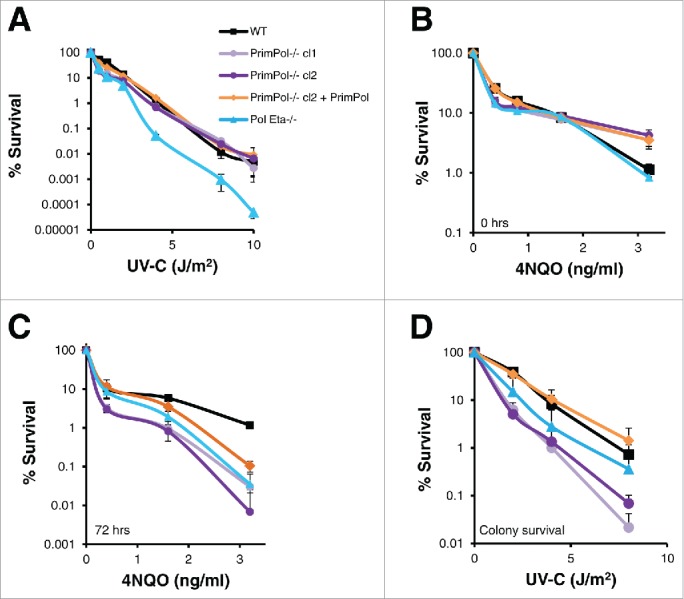
*PrimPol*^*−/−*^ cells show decreased UV-C sensitivity with dose compared to wild type and *Pol* η^*−/−*^ in viability but not clonal survival assays. (A) Cell viability was measured after increasing doses of UV-C (48 hrs after damage) using Cell Titer Blue, lines represent an average of 3 repeats. (B) Cells were grown in the presence of increasing doses of 4NQO or media alone for 48 hrs followed by viability analysis with Cell Titer Blue, or after 48 hrs they were washed with PBS and grown for a further 72 hrs in media alone before viability analysis (C). This method was compared with clonal cell survival after UV-C, where cells were plated singularly after increasing doses of UV-C irradiation and growth to form a colony was counted (n = 3) (D). Significance was calculated using a students T-test, at 2 J/m^2^ p < 0.05 WT: *PrimPol*^*−/−*^ cl1, WT: *Pol* η^*−/−*^ and *PrimPol*^*−/−*^ cl2 p < 0.01. Error bars represent standard deviation of repeat experiments in all cases.

To study the roles of PrimPol in mammalian replication and damage tolerance, we previously generated a *PrimPol*^*−/−*^ DT40 chicken cell line.^8^ We demonstrated that *PrimPol*^*−/−*^ cells exhibited no additional sensitivity to ionising radiation, but had increased sensitivity to UV-C damage, similar to DT40 cells lacking *Pol* η. However, when sensitivity to a wider range of UV-C doses was analyzed, we observed differences between *PrimPol*^*−/−*^ and *Pol* η^*−/−*^ cells. While the sensitivity of *Pol* η^*−/−*^ cells continued to increase linearly, in comparison to their WT counterparts with increasing UV-C doses, cells lacking PrimPol actually became less sensitive in comparison to WT cells when UV-C doses were increased (Figure 1A). The same effect was visible when viable cells were counted using trypan blue staining after UV-C damage (Figure S1A). In addition, similar results were observed when the sensitivity to the UV mimetic drug 4NQO was tested using the Cell Titer Blue viability assays (Figure 1B and C). When cells were incubated with 4NQO for 48 hrs, *PrimPol*^*−/−*^ cells were found to be less sensitive than WT cells at higher drug doses. However, when cells were washed clear of the drug and allowed to recover for a further 72 hrs, *PrimPol*^*−/−*^ cells became much more sensitive at all doses of 4NQO, in a similar manner to *Pol* η^*−/−*^ cells. Notably, in these assays sensitivity was measured using Cell Titer Blue, which assesses the ability of a cell population to metabolise resazurin but not the proliferative capacity of the cells. Therefore, colony formation assays were employed to measure cell survival and quantify the ability of individual cells to expand to form a viable population following exposure to UV-C damage. *PrimPol*^*−/−*^ cells were found to be much more sensitive to UV-C, at all doses, compared to WT cells and were also more sensitive than cells lacking *Pol* η (Figure 1D). Thus, although more *PrimPol*^*−/−*^ cells remain metabolically active after UV-C damage or 4NQO treatment, they are unable to proliferate to the same extent as WT cells.

### UV-C damage induces increased mitotic defects and decreased cell death in *PrimPol*^*−/−*^ cells

**Figure 2.**
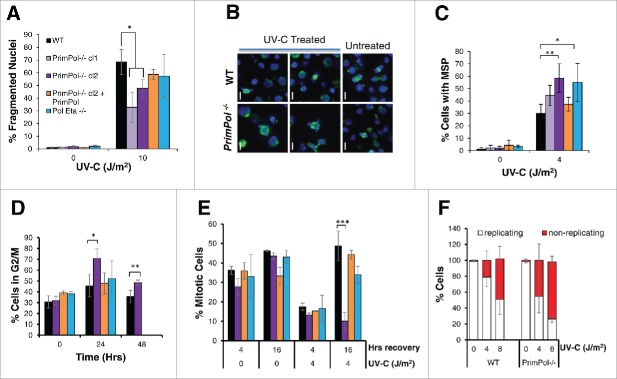
UV-C damage causes extended G2 arrest in *PrimPol*^*−/−*^ cells leading to decreased cell death but increased aberrant mitotic division. (A) Cells were stained with DAPI and normal nuclei populations were compared for the percentage of fragmented nuclei 16 hrs after UV-C damage, n ≥ 3 independent experiments and error bars represent standard deviation. (B) Cells were also co-stained with α-tubulin to identify mitotic cells with multipolar spindles, example images (16 hrs after 2 J/m^2^ UV-C) (scale bar 10 µM). Quantification (16 hrs after 4 J/m^2^ UV-C) is shown in (C). (D) Cells were analyzed by FACS after propidium iodide staining at increasing recovery time-points after 4 J/m^2^ UV-C damage, average G2/M population is shown from 3 independent experiments. (E) Mitotic entry was analyzed by p-H3 staining during a 4 hr nocodozole treatment, 0 or 16 hrs after 0 or 4 J/m^2^ UV-C damage. (F) Cells unable to undergo replication during a 16 hr EdU labeling were counted after 0 or 4 J/m^2^ UV-C followed by a 24 hr recovery period, representative images shown in Figure S2C. In all cases error bars represent standard deviation and significance was measured using an unpaired students T-test (* p < 0.05, ** p < 0.01, ***p,0.001).

Visual inspection of DT40 cells, after treatment with increasing UV-C doses, revealed a large escalation in apparent cell death. To analyze this more closely, cells were stained with 4',6-diamidino-2-phenylindole (DAPI) and fragmented nuclei, indicative of cell death, were counted as a percentage of the total cell population (Figure 2A and S1B). We observed a decrease in cell death in *PrimPol*^*−/−*^ cells, in comparison with WT and *Pol* η^*−/−*^ cells, after high UV-C doses (10 J/m^2^). While at lower UV-C doses (≤4 J/m^2^) the levels of nuclear fragmentation were similar across different cell types (Figure S1C). Therefore, the differences observed in cell viability assays may be explained by a greater frequency of cell death in WT cells and although *PrimPol*^*−/−*^ cells are unable to proliferate, they remain viable. A luciferase-based apoptosis assay further confirmed a difference in cell death after UV-C damage in WT and *PrimPol*^*−/−*^ cells (Figure S1D), revealing a decrease in caspase release in *PrimPol*^*−/−*^ cells after UV-C damage, compared with WT cells, supporting the proposed cell death model.

In addition to apoptosis, we also observed an increase in the number of aberrant mitotic cells in DT40 cells 16 hrs after UV-C damage, with chromosomes segregated in multiple directions. Staining these cells with α-tubulin to identify multiple spindle poles (MSP) revealed a significant increase in defective mitotic cells following UV-C treatment for both *PrimPol*^*−/−*^ and *Pol* η^*−/−*^ cells compared with WT cells (Figure 2B and C). This MSP phenotype could be complimented by the expression of WT PrimPol in *PrimPol*^*−/−*^ cells, thus showing that this increase in MSP is a consequence of loss of PrimPol. Analysis of these MSP over time revealed that levels were similar in both WT and *PrimPol*^*−/−*^ cells at early time points (8 hrs after damage) but MSP levels continued to rise in *PrimPol*^*−/−*^ cells up to ∼16 hrs (Figure S2A). However, levels decreased ∼24 hrs after damage in both cell lines, suggesting that the cause of the multiple spindle poles had been or begun to be resolved. Multiple spindle poles have been attributed to Chk1 expression and may be connected to the prolonged G2 arrest.^25-27^

### UV-C damage induces an extended G2 arrest in *PrimPol*^*−/−*^ cells

After UV-C damage, we observed a significant increase in the size of the DT40 cells. However, while WT cells returned to normal over time, this increased size persisted in *PrimPol*^*−/−*^ cells at least 48 hrs after damage (Figure S2B). This increase in cell size is likely indicative of a G2 arrest. Therefore, we next analyzed cell cycle profiles of *PrimPol*^*−/−*^ cells in more detail by flow cytometry. These cells showed a significant increased population in G2/M in comparison with WT cells (Figure 2D), and in contrast to *Pol* η^*−/−*^ cells, they could be complemented by expression of Hs PrimPol in *PrimPol*^*−/−*^ cells. To follow this G2/M arrest in more detail, entry into mitosis was quantified in the presence or absence of UV-C damage. Cells were first treated with 0 or 4 J/m^2^ UV-C and, at 0 or 16 hrs after damage, nocodazole was added to prevent exit from mitosis. The number of cells entering mitosis within 4 hrs was analyzed by phospho-H3 staining (Figure 2E). Little difference was observed between any of the cell lines in the first 4 hrs after damage. However, a striking block to mitotic entry was observed in *PrimPol*^*−/−*^ cells 16-20 hrs after UV-C damage, which was not observed in *Pol* η^*−/−*^ cells or *PrimPol*^*−/−*^ complimented with Hs PrimPol. To confirm that these G2 cells become arrested for a prolonged period, in a non-replicating / proliferating quiescent state, we performed long-term EdU labeling to identify actively cycling cells. Cells were treated with 0 or 4 J/m^2^ UV-C and allowed to recover for an increasing amount of time before being labeled with EdU for 16 hrs, approximately 2 cell cycles so all cells should label at least once if they are actively proliferating. When a short recovery time was applied, 2 hrs followed by EdU labeling, virtually all of the cells were found to be EdU positive, (data not shown). However, 24 hrs after recovery followed by 16 hrs EdU labeling, a large population of unlabelled cells were observed in *PrimPol*^*−/−*^ cells (Figure 2F and S2C). Together, these results establish that, following significant UV-C damage, *PrimPol*^*−/−*^ cells become arrested in G2 for an extended period and are prevented from progressing into mitosis. These findings are in contrast with cells lacking another TLS polymerase, Pol η, which displayed a modest decrease in entry into mitosis after UV-C damage. This increased and extended G2 arrest exhibited by *PrimPol*^*−/−*^ cells explains earlier reported differences in their UV-C damage sensitivities.

### Extended G2 arrest is partially dependent on Chk1

**Figure 3.**
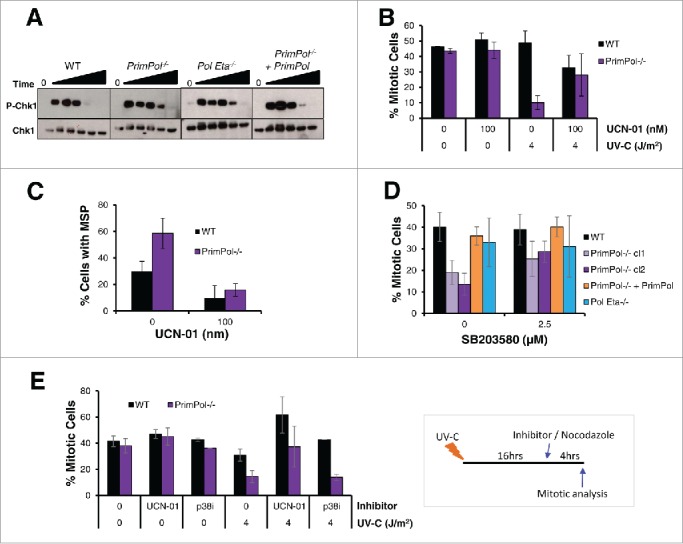
UV-C induced checkpoint activation in *PrimPol*^*−/−*^ cells is partially resolved by inhibition of Chk1 or p38. (A) Chk1 phosphorylation was analyzed by western blotting of whole cell lysates at increasing recovery times (2-24 hrs) after 4 J/m^2^ UV-C damage. (B) The affect of UCN-01 on cell cycle progression was measured by counting the presence of p-H3 positive mitotic cells. Cells were pre-treated with 100 nM UCN-01 for approximately 2 hrs before irradiation with 0 or 4 J/m^2^ UV-C, cells were allowed to recover for 0 or 16 hr before the addition of nocadozole to block mitotic exit for 4 hrs. (C) Mitotic segregation was analyzed by staining with DAPI and α-tubulin 16 hrs after cells were damaged with 4 J/m^2^ in this case cells were pre-treated and then maintained in 100 nM UCN-01 prior to damage. (D) Effect of p38 on cell cycle progression was measured by counting the percentage of p-H3 positive mitotic cells 4 hrs after incubation with nocodazole. Cells were first pre-treated with 2.5 μM SB203580 for 2 hrs followed by irradiation with 4 J/m^2^ UV-C, and a 16 hr recovery period. (E) The ability of checkpoint inhibitors to release cells from G2 arrest was measured by allowing cells to recover after 0 or 4 J/m^2^ UV-C for 16 hrs, followed by addition of 100 nM UCN-01 or 2.5 µM SB203580 and 0.5 µM nocodazole for 4 hrs. Mitotic entry was then assessed by p-H3 staining. For all experiments n ≥ 3 independent experiments, error bars represent standard deviation.

One of the best studied regulators of the G2/M checkpoint is the Chk1 kinase. Chk1 is phosphorylated after damage recognition by the upstream kinases ATR and ATM and the activated kinase prevents exit from G2 into mitosis until the checkpoint is lifted after damage repair.^28,29^ To examine the causes of the enhanced G2 arrest in *PrimPol*^*−/−*^ cells, the level of Chk1 phosphorylation was analyzed at increasing time points after 4 J/m^2^ UV-C damage by western blot of whole cell lysate (Figure 3A). Surprisingly, although Chk1 phosphorylation was somewhat increased in *PrimPol*^*−/−*^ cells, for a longer period up to 16 hrs, these changes appear unlikely to be sufficient to explain the significant arrest apparent in these cells. The role of Chk1 in the G2 arrest of *PrimPol*^*−/−*^ cells was probed specifically using the Chk1 inhibitor UCN-01. *PrimPol*^*−/−*^ and WT cells were pre-treated with the inhibitor prior to UV-C damage and mitosis entry and multipolar spindles were analyzed as before. The addition of the Chk1 inhibitor made little difference to mitotic entry in the absence of damage however, a significant increase in *PrimPol*^*−/−*^ cells entering mitosis was observed 16-20 hrs after UV-C damage (4 J/m^2^) (Figure 3B). Yet, the percentage of mitotic cells did not reach levels observed in undamaged cells. Moreover, a decrease was observed in WT cells after UCN-01 treatment, which is likely due to increased levels of cell death caused by the inhibitor.

While the Chk1 inhibitor was unable to fully restore the mitotic entry levels observed in undamaged cells, it did significantly abolish the numbers of mitotic cells with multipolar spindles in both WT and *PrimPol*^*−/−*^ cells (Figure 3C). Therefore, these multipolar spindles are caused by activation of Chk1 directly or the G2/M checkpoint and its extension (Figure 2C), rather than the direct loss of PrimPol itself.

### Role of p38 in the extended G2 arrest after UV-C damage

The p38 MAP kinase pathway has also been implicated in the activation of the G2/M checkpoint. p38 is thought to be activated by damage in G2 cells when there is an absence of single-stranded (ss) DNA.^30-33^ As a limited increase in ssDNA breaks was observed in *PrimPol*^*−/−*^ cells after UV-C damage (Figure S2 D, E, F), we examined the impact of the p38 kinase using the SB203580 inhibitor. Cells were pre-treated with p38 inhibitor followed by 4 J/m^2^ UV-C and mitotic entry analyzed 16-20 hrs after damage (Figure 3D). Notably, *PrimPol*^*−/−*^ cells showed a significant increase (P < 0.0015) in mitotic cells after pre-treatment with SB203580 (2.5 μM), while there was little change in the mitotic populations in WT and *Pol* η ^*−/−*^ cells or those complimented with Hs PrimPol. However, unlike with the Chk1 inhibitor, addition of SB203580 did not cause release from the checkpoint, when added 16 hrs post damage (Figure 3E). This suggests that p38 plays a role in the initiation of the G2/M checkpoint in some *PrimPol*^*−/−*^ cells after UV-C damage, but does not appear to be responsible for keeping them in G2 for an extended period of time.

### *PrimPol*^*−/−*^ cells are resistant to cell death caused by checkpoint inhibition

**Figure 4.**
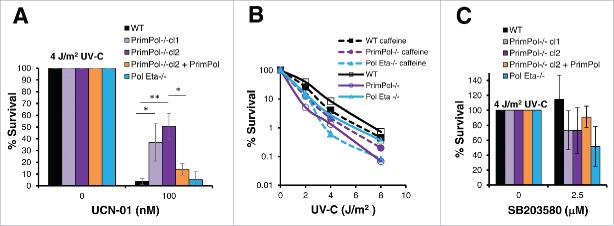
*PrimPol*^*−/−*^ cells are more resistant to Chk1 inhibition than WT cells. (A) Cell viability was measured using Cell Titer Blue, 48 hrs after 4 J/m^2^ UV-C damage, where cells were maintained in 100 nM UCN-01. (B) Colony formation was analyzed in the presence of 2 mM caffeine after increasing doses of UV-C damage in comparison with survival in the absence of the inhibitor. (C) Cell Titer Blue was used to measure cell viability 48 hrs after 4 J/m^2^ UV-C damage, where cells were pre-treated and maintained in 2.5 µM of SB203580, p38 inhibitor. Error bars represent standard deviation of independent repeats and significance was analysed using a students T-test (*p < 0.05, **p < 0.01).

Surprisingly, when cell survival was analyzed after Chk1 inhibition, *PrimPol*^*−/−*^ cells showed a greater viability in both the presence and absence of UV-C damage. Cells were first pre-treated with 100 nM UCN-01, before 0 or 4 J/m^2^ UV-C damage, and allowed to recover for 48 hrs in the presence of the UCN-01 inhibitor. Cell viability analysis showed a significant improved survival in cells lacking PrimPol, this improvement was not observed when cells were complimented with Hs PrimPol, nor was such an improved survival seen in the absence of Pol η (Figure 4A and S3A). In addition, improved survival was also observed with an ATR inhibitor (NU6027) or the ATR and ATM kinase inhibitor caffeine (Figure S3B, C, D and E). Thus, this affect is a direct response to the inhibition of the checkpoint response.

As described previously, viability assays can mask underlying differences in the survival response. We therefore used colony formation assays to assess survival after UV-C damage in the presence of checkpoint inhibition. Caffeine was added to inhibit the ATR and ATM kinases and we observed that *Pol* η^*−/−*^ cells were now more sensitive to UV-C damage than *PrimPol*^*−/−*^ cells following inhibition of the G2/M checkpoint (Figure 4B, S3F). While both WT and *Pol* η^*−/−*^ cells showed a decrease in survival in the presence of caffeine, *PrimPol*^*−/−*^ cells exhibited greater survival. Thus, activation of the G2/M checkpoint in *PrimPol*^*−/−*^ cells appears to be more stringent than may be necessary.

However, the same affect was not observed when we analyzed survival after inhibition of the p38 kinase. In this case, *PrimPol*^*−/−*^ cells actually became less viable after UV-C damage in the presence of the p38 inhibitor, in a similar manner to *Pol* η^*−/−*^ cells, while little affect was noted in WT cells (Figure 4C, S3G).

**Figure 5.**
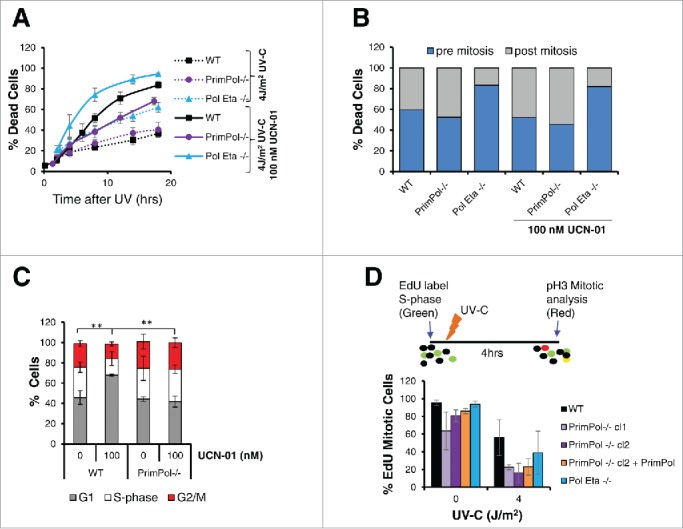
Decreased rates of cell cycle progression are protective in *PrimPol*^*−/−*^ cells. (A) Cell death over time was followed by bright field live cell imaging of H2B RFP labeled cells (WT and *PrimPol*^*−/−*^), after 4 J/m^2^ UV-C damage with or without the presence of 100 nM UCN-01 by counting dead cells as a percentage of the whole population. (B) DT40 cells were followed by live cell imaging after UV-C, allowing the point of cell death to be observed. Cell death was quantified dependent on whether the cell had undergone mitosis prior to death. (C) The effect of the UCN-01 inhibitor on cell cycle populations was analyzed by flow cytometry on propidium iodide stained cells after 24 hrs incubation with 100 nM UCN-01 in the absence of damage. (D) Cell cycle progression rates were measured by analysis of the number of S-phase cells marked by an EdU pulse that were able to progress into mitosis, identified by p-H3 staining in a 4 hr period after 0 or 4 J/m^2^ UV-C damage. Error bars represent standard deviation of independent repeats and significance was analysed using a students T-test (*p < 0.05, **p < 0.01).

To study these survival changes in more detail, cells were visualised by live cell imaging after UV-C damage in the presence or absence of UCN-01 inhibitor. In the absence of inhibitor, levels of cell death were similar in WT and *PrimPol*^*−/−*^ cells, up to 18 hrs after 4 J/m^2^ UV-C damage, with higher levels in *Pol* η^*−/−*^ cells. However, when cells were pre-treated with and maintained in 100 nM UCN-01 after UV-C damage, levels of cell death in *PrimPol*^*−/−*^ cells became significantly less than in WT cells (Figure 5A). Thus, although cell death increased in all cell types after the addition of the Chk1 inhibitor, this change was smaller in cells lacking PrimPol and may explain their improved survival outcome.

Strikingly, when the timing of death was analyzed within the cell cycle, a clear difference was found in *Pol* η^*−/−*^ cells. Here, the majority of cell death occurred pre-mitosis, while in *PrimPol*^*−/−*^ and WT cells death pre and post-mitosis death levels were similar (Figure 5B). Notably, the addition of a checkpoint inhibitor made little difference to the timing of cell death in relation to mitosis (Figure 5B).

### Decreased proliferation rates are protective in the absence of PrimPol

To understand the causes of these changes in death and survival in *PrimPol*^*−/−*^ cells in the absence of a G2 checkpoint, we more closely examined the affects these inhibitors had on the cell cycle. When undamaged WT cells were incubated with 100 nM UCN-01 for 24 hrs, a significant change in the cell cycle profile was observed by flow cytometry (Figure S4A). A significant increase in the G1 cell population was observed in WT cells however, little change was observed in *PrimPol*^*−/−*^ cell populations after UCN-01 treatment (Figure 5C). In addition, a significant increase in the sub-G1 population, indicative of dead or dying cells was observed in WT cells, in comparison with those lacking PrimPol, consistent with results from live cell imaging (Figure S4B). Therefore, in addition to Chk1-initiated G2/M checkpoint, other processes are slowing progression through G2 in cells lacking PrimPol.

Previously, we identified a small decrease in doubling time in *PrimPol*^*−/−*^ cells.^8^ A slowing of progression through the cell cycle may act to prevent the increased G1 population in cells after checkpoint bypass due to Chk1 inhibition, therefore we next examined progression through G2 in more detail. Cells were first labeled with EdU, to mark those in S-phase, followed by 0 or 4 J/m^2^ UV-C. Cells were grown for a further 4 hrs in the absence of EdU label and cells, which had reached mitosis during this time, were identified by phospho-H3 staining. As expected, we identified a significant decrease in S-phase cells, which had progressed to mitosis after UV-C damage. However, notably in both the presence and absence of UV-C damage, progression from S-phase to mitosis was slower in *PrimPol*^*−/−*^ cells (Figure 5D). This decrease in progression rates was also slower than that observed in *Pol* η^*−/−*^ cells and may, in part, explain the observed increased survival of *PrimPol*^*−/−*^ cells after checkpoint inhibition.

## Discussion

### PrimPol and the G2 checkpoint

**Figure 6.**
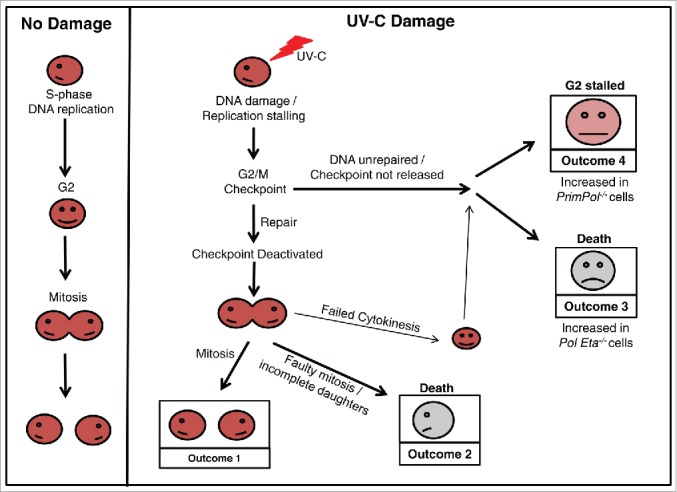
Cell cycle progression in verterbrate cells after UV-C damage. A schematic model showing the possible outcomes as a cell progresses through the cell cycle after UV-C damage, in comparison with undamaged cells. Work described here has identified differences in the percentage of cells achieving each outcome, dependent on its complement of TLS polymerases. An increase in Outcome 4 is observed in the absence of PrimPol, while Outcome 3 becomes more prevalent in cells lacking Pol η.

In this study, we report apparent differences in cell survival of *PrimPol*^*−/−*^ DT40 cells after UV-C damage, when studied by 2 different methods. Although *PrimPol*^*−/−*^ cells appeared more resistance when viability is measured using the metabolic viability assay, Cell Titer Blue, *PrimPol*^*−/−*^ cells exhibit a much greater sensitivity after UV-C damage in colony survival assays. We show that these differences are due to an enhanced activation of the G2/M checkpoint in *PrimPol*^*−/−*^ cells, which prevents them from proliferating but allows then to remain viable (Figure 6, Outcome 4). This checkpoint is largely due to activation of the Chk1 pathway. However, a proportion of this enhanced checkpoint activity is maintained by additional mechanisms, including the p38 kinase pathway and a decreased proliferation rate observed in *PrimPol*^*−/−*^ cells. These observations pose many questions about how and why such an enhanced checkpoint activation is observed in the absence of PrimPol.

Despite the enhancement of the G2 checkpoint in *PrimPol*^*−/−*^ cells and the large extent to which this is dependent on Chk1, as evident by its reversal by the Chk1 inhibitor UCN-01, a surprisingly small increase in Chk1 phosphorylation was observed (Figure 3). A number of different phosphorylation sites have been identified on Chk1, which have been shown to be involved in G2/M checkpoint activation, thus it is possible that other regulation sites/modifications may be important for the activation observed here. However, S_345_ has been shown to be the most prominent in checkpoint activation.^34^ Chk1 is an activator of the G2/M checkpoint and works by activating/inhibiting a number of downstream proteins, such as CDC25, Wee1 and p53,^35^ thus prolonged activation of these may explain the prolonged G2/M checkpoint in *PrimPol*^*−/−*^ cells after UV-C damage.

As well as checkpoint activation, Chk1 has also been shown to play an important role in origin firing and replication fork progression.^36^ Thus, these additional roles may also have some degree of impact upon the addition of the Chk1 inhibitor. For example, loss of Chk1 has been shown to lead to excess origin firing and we therefore speculate that, in the absence of PrimPol, origin firing may be constrained to some extent giving greater UCN-01 resistance.

In addition, little increase in ssDNA was observed in *PrimPol*^*−/−*^ cells after damage and no significant increase in chromosome breaks were found. This leads us to question how the checkpoint itself is activated / maintained and why it fails to be turned off. The increased survival observed after addition of checkpoint inhibitors may be partly explained by the decreased proliferation rates having a protective effect, but it also suggests that not all cells arrested in G2 are inviable once the checkpoint is released. For example, in colony survival assays *PrimPol*^*−/−*^ cells were actually less sensitive after the addition of caffeine, confirming that release of the checkpoint is not detrimental in all cells. Thus, the cause of the arrest may not be as serious as the cell perceives and many of these persistently arrested cells may still be able to proliferate, albeit with potential mutagenic consequences. A number of different polymerases are utilized to bypass particular lesions.^3,4^ Therefore, it is probably unsurprising that stretches of ssDNA were not observed in the absence of PrimPol as one would expect another TLS polymerase to be utilized to bypass UV lesions in its absence. We speculate that, as well as bypassing standard UV lesions, PrimPol may have a more specialized role, such as repriming, which cannot be complimented by other TLS polymerases. It may also be the case that in the absence of PrimPol, a specific “mark” signaling checkpoint activation may not be fully removed or resolved, thus preventing the release of the G2/M checkpoint in some cells.

In addition to Chk1, inhibition of p38 was also found to cause an increase in populations of *PrimPol*^*−/−*^ cells progressing into mitosis after UV-C damage. However, this was only observed when the inhibitor was added prior to UV-C damage and could not be used at later time-points to release cells from the checkpoint. p38 activates the G2/M checkpoint in the absence of ssDNA and is thought to be active in G2 cells,^31^ thus the majority of cells stuck in a prolonged G2 arrest are not maintained by p38. These data suggest that a number of cells enter G2/M checkpoint arrest from G2 due to p38 phosphorylation early after damage but, at later time-points, addition of the p38 inhibitor causes no increase in cells entering mitosis, suggesting that p38 activation is not holding them here or that this checkpoint activation cannot be reversed by addition of the inhibitor. In addition, the re-entry into mitosis observed after addition of the Chk1 inhibitor is greater than that observed with the p38 inhibitor, possibly indicating that the majority of cells entering prolonged G2 arrest were damaged during S-phase, consistent with a role of PrimPol in the completion of replication.

In addition, while *PrimPol*^*−/−*^ cells show resistance to Chk1 inhibitors, they were slightly more sensitive to p38 inhibitors. We identified that this increase in resistance is due to a decreased proliferation rate, which is protective in *PrimPol*^*−/−*^ cells. However, a protective role is not observed after p38 inhibition. This may be due to the different cell cycle roles of the 2 kinases, again suggestive of PrimPol's role in the completion of S-phase. However, it may also be due to the robust activation of the Chk1 checkpoint, such that damage never reaches G2 and thus p38 is not required unless cells are directly damaged in G2.

### PrimPol and Pol η have complimentary roles in tolerance of UV damage

We have identified clear differences in the cellular consequences resulting from the loss of 2 major TLS polymerases, PrimPol and Pol η, following UV-C damage. The fact that a clear sensitivity to UV-C damage was observed in the absence of either polymerase alone confirms that they are unable to fully compliment for the loss of the other, highlighting their distinct roles, activities and substrate specificities in the damage tolerance process. These distinct roles are also evident in human cells where *PrimPol*^*−/−*^ cells, unlike in *Pol* η^*−/−*^ cells, show little apparent sensitivity to UV-C. However, these cells become increasingly sensitive to UV damage when both polymerases are knocked out / down,^8^ again supporting their complmentary roles in UV damage tolerance. The enhanced phenotypes observed for *PrimPol*^*−/−*^ DT40 cells are likely due to their S-phase cell cycle poise, as opposed to G1 in mammalian cells, reflecting their significantly elevated rates of genome replication. Such differences in phenotypic penetrance in avian cells has also been observed in other cases, such as deletion of FancJ.^37^

Live cell imaging has identified key differences in the response to UV-C damage in *PrimPol*^*−/−*^ and *Pol* η^*−/−*^ cells. By following these cells after damage, we found that ∼60 % of cell death occurred in WT cells prior to mitotic division, likely due to accumulation of inviable levels of damage that could not be repaired to relieve the G2 checkpoint thus cells undergo apoptosis instead of entering mitosis. In *PrimPol*^*−/−*^ cells, this decreased to ∼50 % cell dead. In striking contrast, this value was increased to >80 % of cells in Pol η^−/−^ cells (Figure 5B). Therefore, the resulting consequences of TLS polymerase absence are different in both cases (Figure 6). When Pol η is absent then damage becomes unrepairable and leads to cell death, while in the absence of PrimPol the outcomes are significant enough to stall the release of the G2 checkpoint, but do not trigger cell death until after division. In addition, a number of tripolar mitotic events and cytokinesis defects were observed (Figure S4C), which may provide clues about the possible causes of death in these cells.

Previous studies of *Pol* η^*−/−*^ cells reported an increase in ssDNA after UV-C damage.^38^ However, this was not evident in *PrimPol*^*−/−*^ cells suggesting that the cause of the sensitivity and enhanced checkpoint activation may be more complex than simply an inability to fill in gaps left at UV-C induced lesions, a role which Pol η can undertake even in the absence of PrimPol. Therefore, we speculate that PrimPol may be involved in the bypass or, more likely, re-priming at specific structures or lesions, which cannot be fully complimented by Pol η. Alternatively, another aspect of UV-C damage repair fails to be completed sufficiently to allow release of the G2 checkpoint and subsequent progression into mitosis.

### PrimPol and cell death following UV-C damage

Several studies indicate that S-phase progression is important for triggering apoptosis, independently of the repair capacity of the cells.^1,39-41^ Therefore, the decreased apoptosis observed in *PrimPol*^*−/−*^ cells irradiated with high UV-C fluences could be a direct consequence of a delay in S-phase progression caused by the absence of PrimPol.^8^ Furthermore, high fluence exposures induce an increased number of photoproducts leading to a stronger activation of the checkpoint, thus resulting in inhibition of both DNA replication initiation and elongation.^42-44^ Our data support a model in which the absence of PrimPol, specifically after high UV-C doses exposure, further delays DNA replication elongation, reinforcing the checkpoint response and thus further decreasing the apoptosis response. However, following a lower dose of UV-C irradiation, below the threshold responsible for a strong checkpoint activation, cells rely more on PrimPol to bypass or restart stalled replication forks in a faster but potentially more inaccurate manner,^24^ instead of activating a cell cycle arrest and triggering a much slower DNA repair pathway response. This pro-apoptotic role of a DNA damage tolerance factor is not unprecedented as Pol η has also been demonstrated to be pro-apoptotic following HU treatments.^45^ de Feraudy *et al.* concluded that this decrease of apoptosis was due to a cell cycle delay in S-phase (due to the absence of the TLS polymerase), resulting in a slower rate of XPV cells to reach the G1/S boundary responsible for this HU-induced apoptosis response.^45^ A similar phenotype was observed here in the absence of PrimPol following UV-C irradiation, leading to an extended G2 arrest and a decrease in apoptosis (Figure 6). Further studies are required to elucidate the additional causes of this extended G2/M checkpoint and why, in the absence of PrimPol, this is not released in a number of cells, yet these cells do not undergo cell death. A greater understanding of the precise roles played by PrimPol in damage tolerance, via its TLS and re-priming functions, will allow us to decipher the consequences of its loss for genome replication and cell viability after damage.

## Materials and methods

### Cell lines and drug treatment

DT40 cells were grown in RPMI supplemented with 10 % fetal calf serum, 1 % chicken serum, 1 % penicillin streptomycin, 1% L-glutamine and 10 μM β-mercaptoethanol. PrimPol knockout and complimented cell lines were generated as described previously.^8,12^ Cells were pre-incubated with all checkpoint drugs for 2 hrs prior to damage, UCN-01 (Sigma), SB203580 (Sigma), caffeine (Sigma), NU6027 (Sigma). Cells were then resuspended in a smaller volume of PBS and irradiated with UV-C before media containing the same concentration of inhibitor was returned.

### Immunofluorescence studies

Cells to be analyzed by microscopy were grown as normal and then cytospun onto glass slides and fixed with 3 % paraformaldehyde prior to imaging. Cells were either stained directly with DAPI or with antibodies to α-tubulin (Sigma), phospho H3 (Abcam), followed by alexa-fluoro labeled secondary antibodies (Invitrogen), EdU was visualised using the “Click-it” reaction (Invitrogen). Cells were imaged on a wide-field DeltaVision Olympus IX70 microscope or counted on a Nikon E400 microscope.

### Cell cycle analysis

Cell cycle populations were measured using flow cytometry on a BD FACSCanto machine (BD). Approximately 5 × 10^6^ cells were collected and fixed in 70 % ethanol. Cells were then stained with 10 μg/ml propidium iodide and 0.5 mg/ml RNase A in PBS. Results were analyzed using FACSDiva software (BD).

To monitor cell cycle progression timing cells were first labeled with 10 µM Edu for 20 minutes. They were then washed clean of this label in PBS and treated with UV-C if required. At desired times after this cells were collected, cytospun onto slides and fixed with 3 % paraformaldehyde. Cells were stained with p-H3 antibody and fluorescent secondary antibody and Edu was detected using the “Click-It” kit (Invitrogen). Slides were imaged on a DeltaVision Olympus IX70 microscope and the numbers of positive cells counted using Image J software.

To analyze the effects of checkpoint inhibitors on cell cycle, DT40 cells were pre-treated for 2 hrs with the relevant inhibitors before being resuspended in PBS and exposed to 0 or 4 J/m^2^ UV-C. Media was then replaced with the same inhibitor concentrations and the addition of 1 µM nocodazole to block mitosis exit. At 4 hrs after damage cells were collected and stained as above for phospho-H3 to allow percentage of mitotic cells to be counted.

### Cell survival assays

∼1×10^4^ cells (or a serial expansion were survival was found to be much lower) were diluted in a small volume of PBS and treated with required dose of UV-C, followed by the addition of standard media. Cells were then grown for the stated time and 100µl were transferred to a 96 well plate. Cell Titer-Blue (Promega) a metabolic capacity substrate, was added and viability assayed on a Glomax plate reader.

For colony survival assays, 200 cells (or a multiple of this dependent on expected survival) were treated with UV-C as above and then diluted into 20 ml media and plated in 2 x 96 well plates. Cells were grown for a 1-2 weeks and the presence of a colony was counted by eye and corrected for cells plated.

Caspase activation was measured using Caspase Glo (Promega) following 8 hrs recovery after UV-C treatment of the cells.

### Western blotting

Whole cell lysate was collected at the desired times with or without prior damage. 30 µg total protein was separated by SDS PAGE and protein gel blotted. Proteins of interest were detected by antibodies to S_345_P-Chk1 (Cell Signaling), α-tubulin (Sigma), Chk1 (Santa Cruz) and Hrp labeled secondary (Abcam and Dakko).

### Comet assays

Comet assays were carried out as described.^46^ Briefly cells were resuspended in PBS and treated with 0, 60, 120, 240 J/m^2^ UV-C before being returned to standard media and allowed to recovery for 2 hrs. 3×10^4^ cells were washed with PBS and resuspended in 0.6 % agarose and set upon an agarose cushion. Cells were denatured in NaOH lysis buffer, pH10 for 1 hr and then separated by electrophoresis. Cells were stained with Sybr green and analyzed using Comet software on a Nikon E400 microscope.

### Chromosome spreads

DT40 cells were incubated with 1 μM nocodazole for 4 hrs either directly or 16 hrs after UV-C treatment. Cell were swollen with 75 mM KCl and fixed with 3:1 methanol : acetic acid before being dropped onto glass slides. Slides were fixed with methanol and stained with Giemsa (Sigma) before being imaged on a wide-field DeltaVision Olympus IX70 microscope.

### Live cell imaging

DT40 cells stably transfected with RFP labeled H2B (WT and *PrimPol^−/−^*) were used for live cell imaging. Imaging was carried out using an Olympus IX71 microscope with a 40 x lens. Cells were maintained at 37°C in the presence of 5 % CO_2_ during imaging in standard RPMI media and images were taken at 10 minute intervals over 16 hrs. Cells were pre-treated with inhibitors as for fixed imaging and then UV-C treated in imaging dishes before being allowed to settle for 2 hrs in a standard incubator prior to image acquisition.

## Supplementary Material

1128597_Supplemental_Material.pdf
