# Supplementary material for: PrimPol-deficient cells exhibit a pronounced G2 checkpoint response following UV damage
Source: Cell Cycle. 2015 Dec 22;15(7):908–18. doi: 10.1080/15384101.2015.1128597 (PMC4889237; doi:10.1080/15384101.2015.1128597)
Supplement: 1128597_Supplemental_Material.pdf [file kccy-15-07-1128597-s001.pdf]

Figure S1. *PrimPol$^{-/-}$* DT40 cells show altered survival and morphology after UV treatment. (A) Live cells were counted on a haemocytometer with trypan blue stain 48 hrs after increased doses of UV-C. (**B)** Representative images of DAPI stained cells showing an increase in cells with fragmented nuclei 16 hrs after increasing UV-C doses, quantified in Fig 2A, scale bar 10 μm. **(C)** Quantification of cells with fragmented nuclei 16 hrs after lower UV-C doses. **(D)** Apoptosis was analysed by following the activation of caspase 3 using a luminescence assay (Promega). WT and *PrimPol$^{-/-}$* cells were analysed at 8 hrs following increasing doses of UV damage n=3 and error bars represent standard deviation.

Figure S2. After UV-C treatment a population of cells become enlarged and unable to replicate but display no significant increase in DNA breaks. Mitotic cells with multipolar spindles were counted as a percentage of the population at increasing time-points after 4 J/m^2 UV-C **(A).** DT40 cells were viewed under a light microscope 48 hrs after 5 J/m^2 UV-C damage to examine changes in morphology **(B).** These changes were seen more clearly after staining with DAPI, when cells were treated with 0 or 4 J/m^2 UV-C damage and allowed to recover for 24 hrs before being labelled with EdU for 16 hrs (green) to identify those not undergoing replication, quantified in Fig. 2F, scale bar represents 100 μm **(C).** ssDNA gaps were analysed by alkaline comet assay after increasing doses of UV-C damage **(D).** Cells carrying chromosome breaks were counted in giemsa stained chromosome spreads from cells arrested with 0.5 μM nocodozole for 4 hours after a 0 **(E)** or 16 hr **(F)** recovery period following increasing doses of UV-C damage. In all figures, error bars represent standard deviation for n= 3 independent experiments, significance was measured using a students T-test, * $p<0.05$.

Figure S3. *PrimPol$^{-/-}$* cells are more resistant to abolition of the G2 checkpoint. (A)The effect of 100 nM UCN-01 inhibitor on DT40 cells was assessed by Cell Titer Blue after a 48 hr incubation in the absence of damage**.** The effect of upstream kinases ATR and ATM was analysed by following cell viability using Cell Titer Blue 48 hrs after the addition of 10 μM NU6027, ATR inhibitor after 0 **(B),** 4 J/m^2 UV-C **(C)** or caffeine **(D,E)** treated with 0 or 4 J/m^2 UV-C, respectively. **(F)** The effect of caffeine was also studied in a colony formation assay with 2 mM caffeine. This is the

figure shown in Fig. 4B with addition of error bars representing standard deviation. **(G)** Cell viability in the presence of the p38 inhibitor was quantified 48 hrs after addition of 2.5 μM inhibitor in the absence of damage using Cell Titer Blue.

Figure S4. UCN-01 causes an increase in G1 populations in WT but not *PrimPol*$^{-/-}$ cells. **(A)** FACS analysis was used to assess the effect of the UCN-01 checkpoint inhibitor on undamaged cells by propidium iodide staining 24 hrs after incubation, FACS plots are shown in comparison with untreated cells**,** which are quantified in Fig.5C. **(B)** Sub G1 cells were also quantified, n=3 error bars represent standard deviation and significance was determined by students T-test (* $p<0.05$). **(C)** Images representing a number of aberrant observed by live cell imaging after UV-C damage including failure of cytokinesis and incorrect division to form 3 daughter cells.

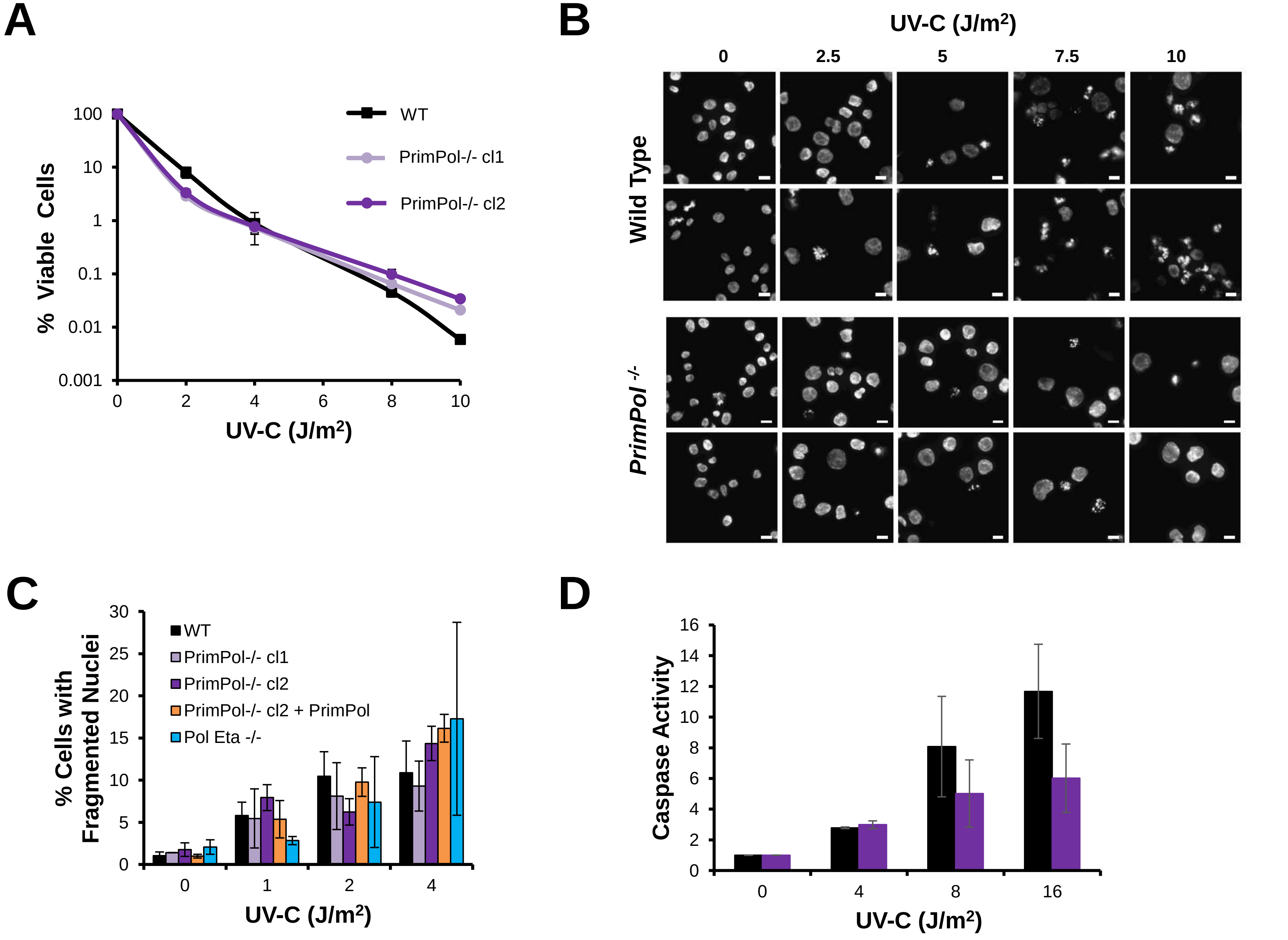
A
100
10
1
0.1
0.01
0.001
% Viable Cells
0
2
4
6
8
10
UV-C (J/m²)
WT
PrimPol-/- cl1
PrimPol-/- cl2
B
UV-C (J/m²)
0
2.5
5
7.5
10
Wild Type
PrimPol -/-
C
% Cells with Fragmented Nuclei
30
25
20
15
10
5
0
WT
PrimPol-/- cl1
PrimPol-/- cl2
PrimPol-/- cl2 + PrimPol
Pol Eta -/-
0
1
2
4
UV-C (J/m²)
D
Caspase Activity
16
14
12
10
8
6
4
2
0
0
4
8
16
UV-C (J/m²)

Figure S1

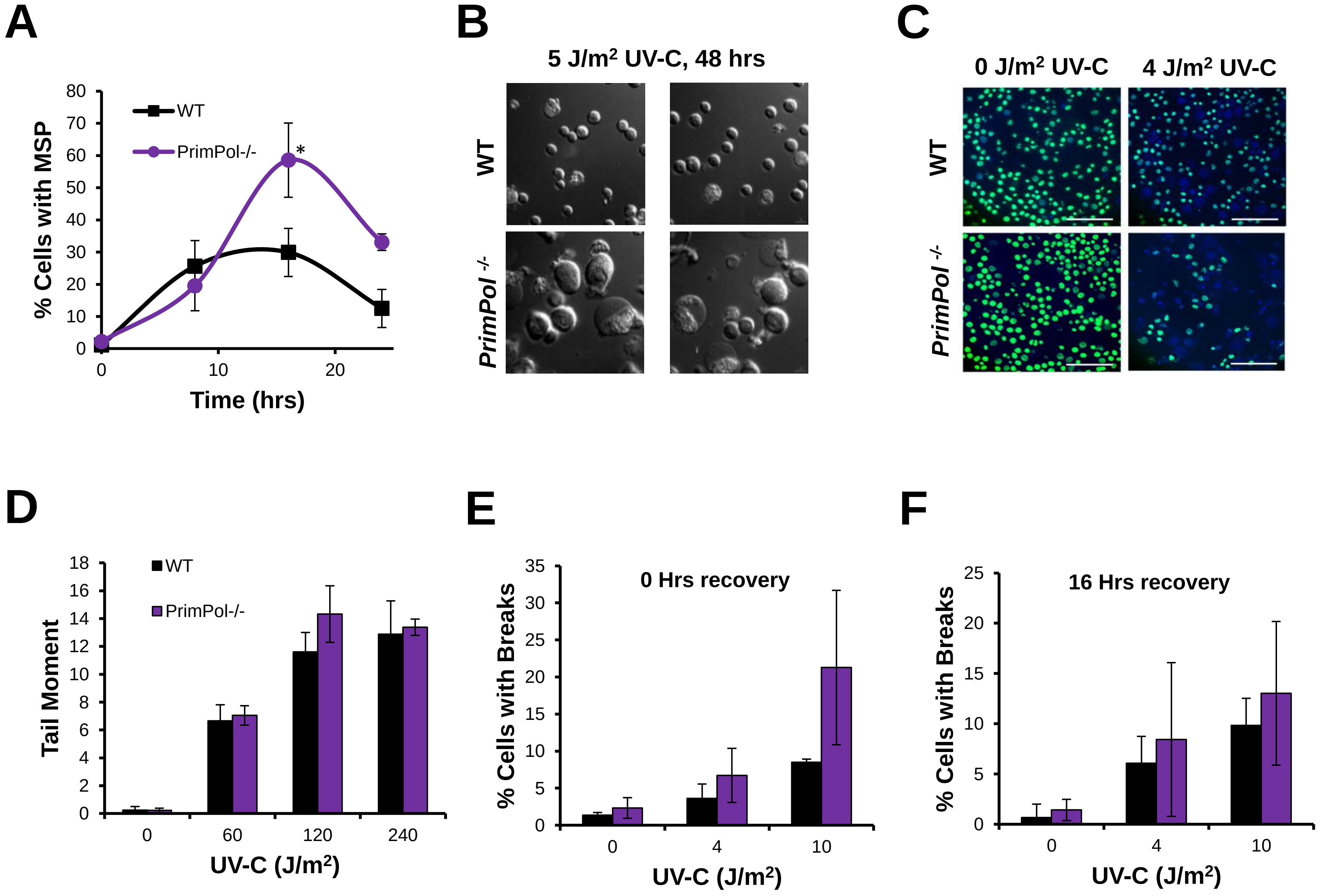

A
80
70
60
50
40
30
20
10
0
% Cells with MSP
WT
PrimPol-/-
*
0
10
20
Time (hrs)
B
5 J/m² UV-C, 48 hrs
WT
PrimPol -/-
C
0 J/m² UV-C
4 J/m² UV-C
WT
PrimPol -/-
D
18
16
14
12
10
8
6
4
2
0
Tail Moment
WT
PrimPol-/-
0
60
120
240
UV-C (J/m²)
E
35
30
25
20
15
10
5
0
% Cells with Breaks
0 Hrs recovery
0
4
10
UV-C (J/m²)
F
25
20
15
10
5
0
% Cells with Breaks
16 Hrs recovery
0
4
10
UV-C (J/m²)

Figure S2

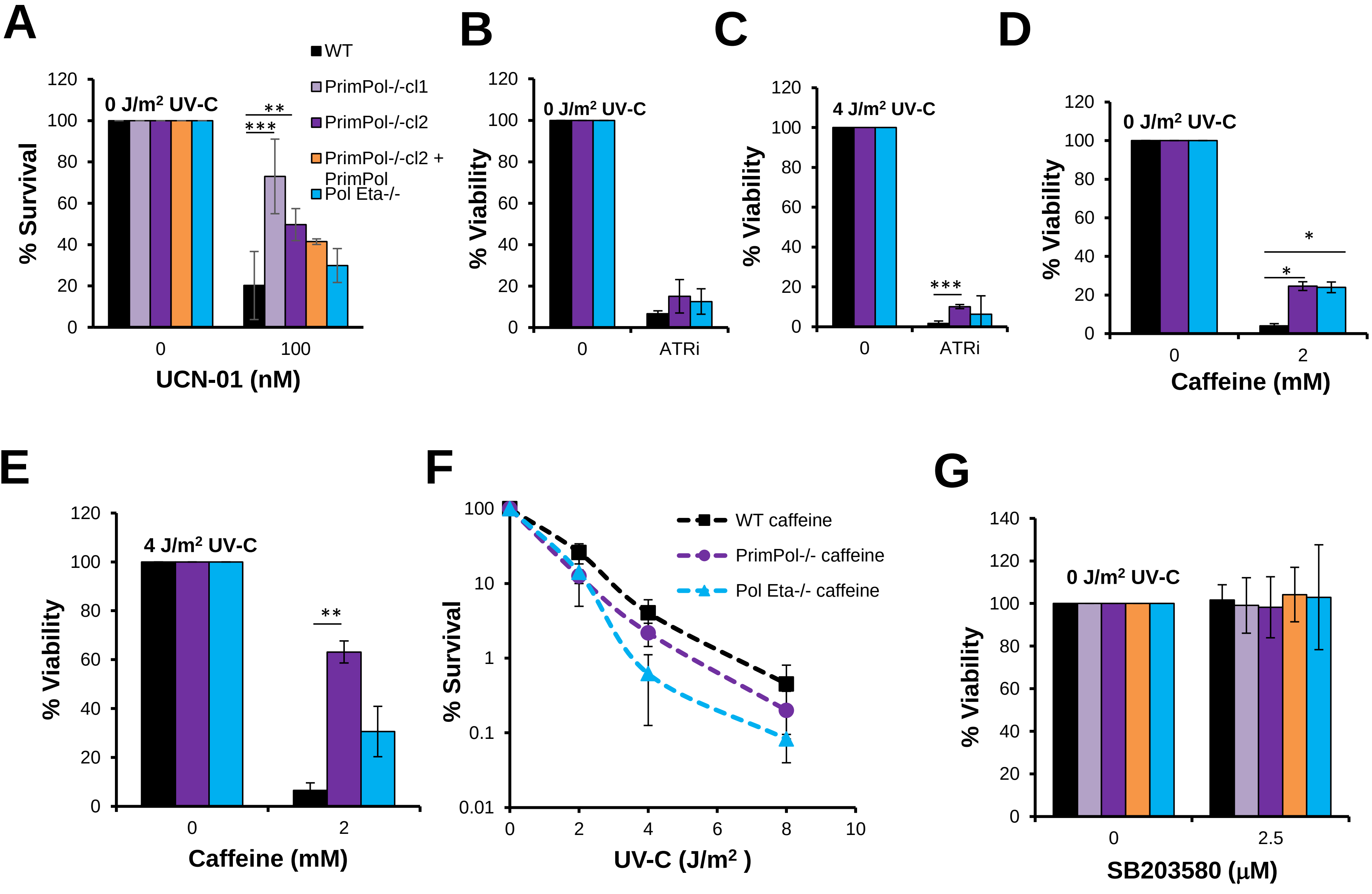
A
WT
PrimPol-/-cl1
PrimPol-/-cl2
PrimPol-/-cl2 + PrimPol
Pol Eta-/-
0 J/m² UV-C
% Survival
UCN-01 (nM)
0
100
**
***
B
0 J/m² UV-C
% Viability
0
ATRi
C
4 J/m² UV-C
% Viability
0
ATRi
***
D
0 J/m² UV-C
% Viability
Caffeine (mM)
0
2
*
*
E
4 J/m² UV-C
% Viability
Caffeine (mM)
0
2
**
F
WT caffeine
PrimPol-/- caffeine
Pol Eta-/- caffeine
% Survival
UV-C (J/m²)
G
0 J/m² UV-C
% Viability
SB203580 (μM)
0
2.5

Figure S3

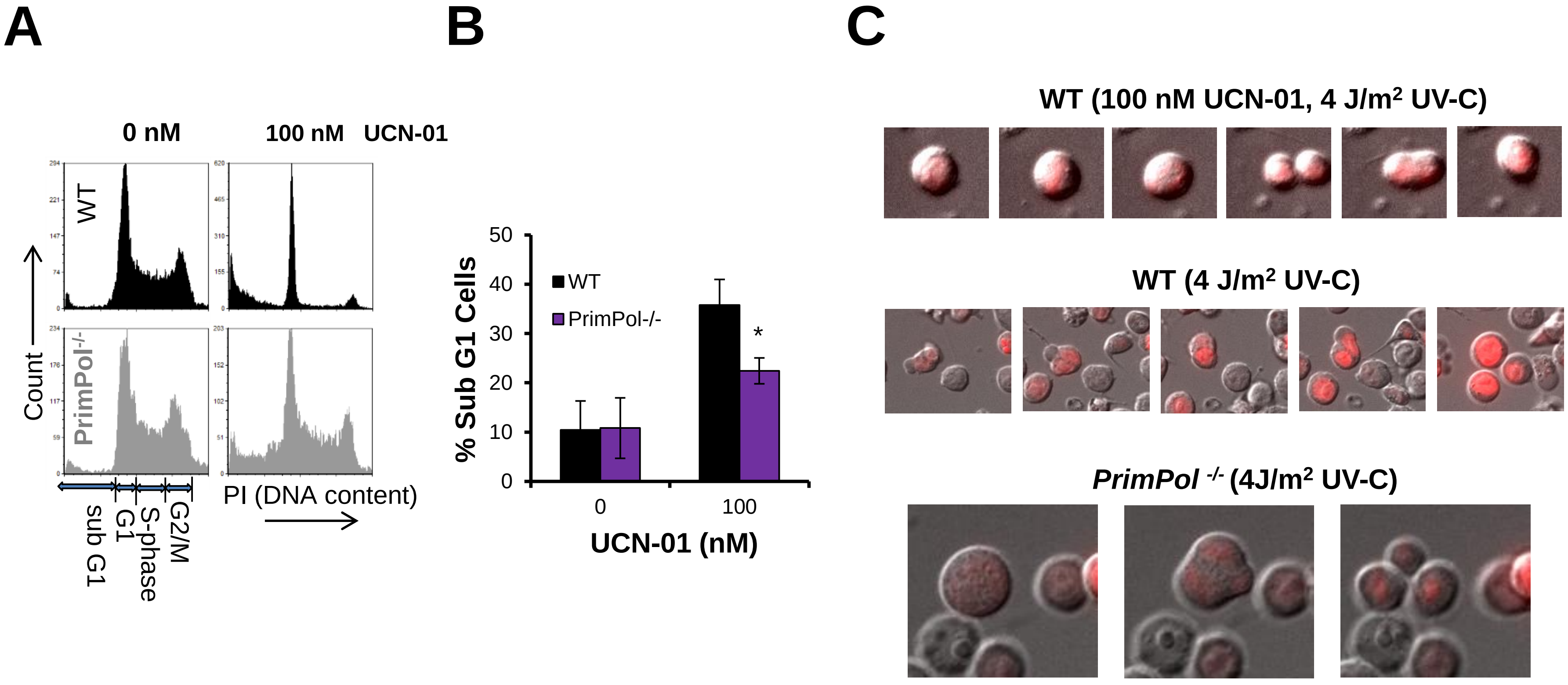
A
0 nM
100 nM UCN-01
WT
PrimPol-/-
Count
sub G1
G1
S-phase
G2/M
PI (DNA content)
B
% Sub G1 Cells
WT
PrimPol-/-
*
0
100
UCN-01 (nM)
C
WT (100 nM UCN-01, 4 J/m2 UV-C)
WT (4 J/m2 UV-C)
PrimPol -/- (4J/m2 UV-C)

Figure S4
